# Molecular interactions of EphA4, growth hormone receptor, Janus kinase 2, and signal transducer and activator of transcription 5B

**DOI:** 10.1371/journal.pone.0180785

**Published:** 2017-07-07

**Authors:** Takahiro Sawada, Daiki Arai, Xuefeng Jing, Masayasu Miyajima, Stuart J. Frank, Kazushige Sakaguchi

**Affiliations:** 1 Department of Molecular Cell Biology and Molecular Medicine, Institute of Advanced Medicine, Wakayama Medical University, Wakayama, Japan; 2 Laboratory Animal Center, Wakayama Medical University, Wakayama, Japan; 3 Department of Medicine, Division of Endocrinology, Diabetes, and Metabolism, University of Alabama at Birmingham, Birmingham, Alabama, United States of America; 4 Birmingham VA Medical Center, Birmingham, Alabama, United States of America; Rutgers University, UNITED STATES

## Abstract

We previously reported that EphA4, a member of the Eph family of receptor tyrosine kinases, is an important modulator of growth hormone (GH) signaling, leading to augmented synthesis of insulin-like growth factor 1 (IGF1) for postnatal body growth. In the present study, we report the molecular interactions of EphA4, GH receptor (GHR), Janus kinase 2 (JAK2), and signal transducer and activator of transcription 5B (STAT5B). EphA4 binds to GHR at both its extracellular and intracellular domains and phosphorylates GHR when stimulated with a ligand. The cytoplasmic domain of EphA4 binds to the carboxy-terminus of JAK2 in contrast to the known binding of GHR to the amino-terminus. STAT5B binds to the amino-terminal kinase domain of EphA4. Ligand-activated EphA4 and JAK2 phosphorylate each other and STAT5B, but JAK2 does not appear to phosphorylate EphA4-bound STAT5B. Ligand-activated EphA4 induces the nuclear translocation of STAT5B in a JAK2-independent manner. GHR expression is required for the activation of STAT5B signaling, even via the JAK2-independent pathway. Various ephrins that have affinity for EphA4 induce STAT5B phosphorylation. These findings suggest the molecular mechanisms by which ephrin/EphA4 signaling enhances the canonical GH-IGF1 axis.

## Introduction

Growth hormone (GH) is an anterior pituitary-derived peptide hormone that exerts a diverse array of physiological actions on body growth and metabolism. The GH-insulin-like growth factor 1 (IGF1) axis plays a major role in postnatal body growth. Growth hormone receptor (GHR) is a member of the cytokine receptor superfamily and is expressed in a variety of cells. GHR, devoid of enzymatic activity, is coupled to Janus kinase 2 (JAK2) [1,2]. GH, when bound to GHR, triggers a conformational change of a preformed GHR dimer at the cell surface [3–5], which is followed by the catalytic activation of JAK2 by transphosphorylation. The best-characterized targets of JAK2 phosphorylation are members of the signal transducer and activator of transcription (STAT) family of transcription factors. The JAK-STAT pathway is regarded as the major effector of GHR signaling [6] and is required for the transcriptional regulation of IGF1 in hepatic cells as well as in many tissues [7,8]. Among several STAT proteins, STAT5B is directly linked to the transcriptional regulation of IGF1 [7,9].

Eph receptors belong to a superfamily of receptor tyrosine kinases classified into 2 subclasses, A and B, by their ligand-binding specificity [10]. EphA receptors bind to a class of ligands called ephrin-As; these ligands are tethered to the cell membrane through a glycosyl phosphatidyl inositol anchor. EphB receptors bind to another class of ligands called ephrin-Bs; these ligands have a transmembrane domain and a short cytoplasmic domain [10–12]. EphA4 binds not only to all ephrin-As but also to ephrin-B2 and ephrin-B3. These Eph receptors interact with several molecules, such as fibroblast growth factor receptors [13], integrins, GTPase-activating proteins, and guanine nucleotide exchange factors, and mediate repulsive axon guidance, angiogenesis, cell migration, boundary formation, and cell growth [12,14–18].

Previously, we reported the role of EphA4 in JAK2-dependent and -independent STAT5B activation that leads to the enhanced synthesis of IGF1 [19]. We found that EphA4, GHR, JAK2, and STAT5B interact physically with each other, and that the eventual crosstalk between the ephrin/EphA4 and the GH/GHR/JAK2 signal transduction pathways augments STAT5B activation by phosphorylation, leading to the enhanced expression of *IGF1* mRNA. However, the detailed molecular interactions of the signaling molecules involved in these pathways have not been clarified yet. In the current study, we extensively examined the molecular interactions among EphA4, GHR, JAK2, and STAT5B using deletion mutants of EphA4, GHR, and JAK2.

## Materials and methods

### Animals

This study was carried out in strict accordance with the recommendations in the Guide for the Care and Use of Laboratory Animals of the National Institutes of Health. The protocol was approved by the Committee on the Ethics of Animal Experiments of Wakayama Medical University (Permit Numbers: 371 and 555). All surgery was performed under sodium pentobarbital anesthesia, and all efforts were made to minimize animal suffering.

### Cells

Mouse embryonic fibroblasts (MEF) with genotypes of wild-type (WT), *Epha4*-/-, and *Ghr*-/- [20] were derived from E14.5 mice using a standard protocol and the genotype was confirmed subsequently. MEF were maintained individually in Dulbecco’s modified Eagle medium (Sigma-Aldrich, Tokyo, Japan; Product #D5796) supplemented with 10% fetal bovine serum (Equitech-Bio, Kerrville, TX, USA). MEF from *Jak2*-/- mice were a gift from Drs. Wagner and Lin, Nebraska Medical Center. Before incubation with human GH or clustered ephrin-A1, the cells were plated onto 6-cm plates at 1.5 × 10^6^ cells/plate. Once at 70–80% confluency, they were pre-incubated overnight with serum-free minimum essential medium (Sigma-Aldrich; Product #M4655) containing 0.5% bovine serum albumin (Sigma-Aldrich; Product #A2058) and incubated with ephrins and/or GH. After washing twice with phosphate-buffered saline (PBS) (Sigma-Aldrich; Product #P5368), the cells were harvested. For knockdown of *Ghr* mRNA, fibroblasts were infected with a lentivirus expressing shRNAs (Santa Cruz Biotechnology, Inc., Santa Cruz, CA, USA; Catalog #sc-40016-v) for *Ghr* mRNA at a multiplicity of infection (MOI) of 10. A lentivirus expressing control shRNA (Santa Cruz; Catalog #sc-108080) was also used for control cells with the same MOI. HEK293T cells were cultured and used for transfection as described previously [21].

### Preparation of nuclear and cytoplasmic extracts

We used NE-PER Nuclear and Cytoplasmic Extraction Reagents (Thermo Fisher Scientific Inc., Yokohama, Kanagawa, Japan; Product #78833) to achieve this purpose. The cells were detached from a dish at the time indicated using a scraper after rinsing with ice-cold PBS.

### Construction of eukaryotic expression vectors and retrovirus expression vectors

Most of the cDNAs for human EphA4 mutants were constructed as described previously [13]. cDNAs for EphA4 mutants with deletion of amino acids 562–760 (EphA4Δ562–760) and the entire cytoplasmic domain (EphA4Δcyto) and for the chimeric EphA4/ephrin-B2 protein were newly generated using a recombinant PCR technique and site-directed mutagenesis. Mouse versions of *Ghr* mutants corresponding to those of the rat [22] were also constructed using the same methods. Mouse *Jak2* mutant cDNAs were constructed as reported [23,24]. The base sequences of all PCR-amplified DNAs were confirmed by sequencing after cloning into a pCRBlunt II-TOPO cloning vector according to the manufacturer’s instructions (Thermo Fisher Scientific Inc.; Product #K280002). For transient eukaryotic expression in HEK293T cells, cDNAs for WT and mutant human *EphA4*, mouse *Jak2*, and mouse *Ghr* were incorporated into a pcDNA3.1 vector (Thermo Fisher Scientific Inc.; Product #V79020) that was modified to express proteins that were carboxy-terminally fused with different peptide tags (3Flag, HA, or 6myc).

For retroviral expression, cDNAs for WT *EphA4*, *Jak2*, and *Ghr* were integrated into the pMXs-IG or pMXs-IP vector. These pMXs vectors (obtained from T. Kitamura, University of Tokyo, Japan) are linked to EGFP or Puro^R^ through the internal ribosomal entry site (IRES) sequence. The pMXs constructs were co-transfected with pCAGVSV-G (encoding vesicular stomatitis virus surface protein under the control of the chicken actin promoter) into HEK293gpIRES cells (obtained from T. Miyazawa, Osaka University, Japan). The resulting pseudotyped retrovirus particles were concentrated by centrifugation at 140,000 × *g* for 180 min. The concentrated retrovirus particles were resuspended in culture medium for the cells to be transduced. Viral transduction was usually carried out at an MOI of 1 or 4. The transduced cells were identified easily by visualization of EGFP fluorescence or selection by puromycin.

### Preparation of ephrins and GH

Ephrin-A1, ephrin-A5, and ephrin-B2 fused to a human IgG-Fc fragment were purchased from Sigma-Aldrich (Tokyo, Japan; Product #E9902, #E0528, and #E0778, respectively). Before application to the cells, 5 μg of these ephrin-Fc compounds were oligomerized by mixing with 12 μg rabbit anti-human IgG-Fc (Jackson ImmunoResearch Laboratories, West Grove, PA, USA; Code #309-001-008) in 0.1 mL PBS at 4°C for at least 1 h. As a control for these ephrin preparations, a human IgG-Fc fragment (Jackson ImmunoResearch Laboratories; Code #009-000-008) was applied after oligomerization. The final concentration of oligomerized ephrins and IgG-Fc used for each study was 0.5 μg/mL. Human recombinant GH (Sigma-Aldrich; Product #S4776) was used at a final concentration of 0.5 μg/mL.

### Transfection, immunoprecipitation, and immunoblotting

Transient transfection with the various expression constructs was carried out using the PerFectin Transfection Reagent (Genlantis, San Diego, CA, USA; Catalog #T303015). The total amount of DNA in each transfection was normalized with the pcDNA3.1 plasmid. HEK293T cells were harvested at 48 h after transfection. MEF were not used for transfection studies since they did not express exogenous proteins effectively. The lysis buffer contained 50 mM HEPES buffer, 1% Triton X-100, 5 mM EDTA, 50 mM sodium chloride, 10 mM sodium pyrophosphate, 50 mM sodium fluoride, 1 mM sodium orthovanadate, and protease inhibitors (1 mM phenylmethylsulfonyl fluoride, 1 μM antipain, 1 μM aprotinin, 1 μM leupeptin, and 1 μM pepstatin A). These reagents were of the highest quality available from the manufacturer (Sigma-Aldrich). In some experiments, cell lysates (25–50 μg) were fractionated directly using sodium dodecyl sulfate-polyacrylamide gel electrophoresis (SDS-PAGE) followed by immunoblotting with an antibody. In other experiments, cell lysates (150–600 μg) were used for immunoprecipitation with an appropriate antibody and protein A-agarose overnight at 4°C followed by washing 3 times with lysis buffer, fractionation by SDS-PAGE, and immunoblotting onto polyvinylidene difluoride membranes (Millipore, Billerica, MA, USA; Catalog #IPVH00010) with an antibody. Immunodetection was performed with the Immobilon Western Blotting Detection System (Millipore; Catalog #WBKLS0500). In some cases, the membranes were stripped and detected with different antibodies. All experiments were carried out at least twice to confirm reproducibility. Quantification of band intensity was carried out on digital data using a computer program (CS Analyzer version 3.0; ATTO Corp., Tokyo, Japan).

The sensitivity and reliability of immunoprecipitation were examined using various eukaryotic expression constructs in HEK293T cells as shown in S1 Fig. Protein A-agarose alone did not immunoprecipitate any protein that could be detected by the secondary antibody for immunoblotting. In some studies, a thick band apparently corresponding to the antibody itself used for immunoprecipitation was detected at a molecular size of approximately 50 kDa, and a few non-specific bands were detected when specific transfectants for protein expression were compared with the vector alone control transfectant. However, the specific protein bands that were exogenously expressed by the transfected DNA constructs were always detectable with the correct antibody. These experiments were repeated to confirm their reproducibility, and the number of repetitions is described in each figure.

### Antibodies

The following antibodies were used in the current study: mouse anti-myc monoclonal (clone 9E10; Santa Cruz Biotechnology; catalog #sc-40); mouse anti-Flag antibody derived from hybridoma (Sigma-Aldrich Co.; FLAG M2; Catalog #F1804); mouse anti-HA monoclonal (Santa Cruz Biotechnology; catalog #sc-7392, marked as anti-HA(m)); rat monoclonal anti-HA high-affinity (Sigma-Aldrich; clone 3F10; catalog #11867423001, marked as anti-HA); rabbit anti-EphA4 polyclonal (Santa Cruz Biotechnology; catalog #sc-921); rabbit anti-GAPDH (Santa Cruz Biotechnology; catalog #sc-25778); mouse anti-phosphotyrosine (anti-pY) monoclonal (Upstate Biotechnology; clone 4G10; catalog #05–321); goat anti-mouse GHR polyclonal (R&D systems; catalog #AF1360); rabbit anti-JAK2 monoclonal (Cell Signaling Technology; catalog #3230); rabbit anti-phospho-JAK2 (Tyr1007/1008) (anti-pJAK2) polyclonal (Cell Signaling Technology; catalog #3771); mouse anti-STAT5B monoclonal (Santa Cruz Biotechnology; catalog #sc-1656); and rabbit anti-phospho-STAT5 (Tyr694) (anti-pSTAT5) polyclonal (Cell Signaling Technology; catalog #9351). The secondary antibodies used for immunoblotting were as follows: goat anti-mouse IgG-HRP (Santa Cruz Biotechnology; catalog #sc-2005); goat anti-rat IgG-HRP (Santa Cruz Biotechnology; catalog #sc-2065); goat anti-rabbit IgG-HRP (Santa Cruz Biotechnology; catalog #sc-2004); and donkey anti-goat IgG-HRP (Santa Cruz Biotechnology; catalog #sc-2065). Selection of the secondary antibody was based on the animal species in which the primary antibody for immunoblotting was produced.

### Reverse transcription quantitative polymerase chain reaction (RT-qPCR)

To quantify the expression levels of *Ghr* mRNA in MEF, cDNA was synthesized with a High-Capacity cDNA Reverse Transcription Kit (Thermo Fisher Scientific Inc.; Product #4368814) and qPCR was performed and analyzed with an iCycler iQ Real-Time PCR Detection System with 1× IQ SYBR Green Supermix (Bio-Rad Laboratories, Tokyo, Japan; Catalog #170–8880) and 5 μM primers. The primer sequences used were as follows: *Ghr*, forward 5′-GATTTTACCCCCAGTCCCAGTTC-3′ and reverse 5′-GACCCTTCAGTCTTCTCATCCACA-3′; and *Gapdh*, forward 5′-ACCCAGAAGACTGTGGATGG-3′ and reverse 5′-GGATGCAGGGATGATGTTCT-3′. The PCR conditions were 95°C for 3 min followed by 40 cycles of 95°C for 10 s and 60°C for 30 s. Relative mRNA expression levels were calculated using the 2^−ΔΔCT^ method to normalize target gene mRNA to *Gapdh* as reported previously [25].

### Statistical analysis

Comparisons between two values were performed by a two-tailed Student’s t test.

## Results and discussion

### GHR binds to EphA4 through extracellular and intracellular domains

**Fig 1:**
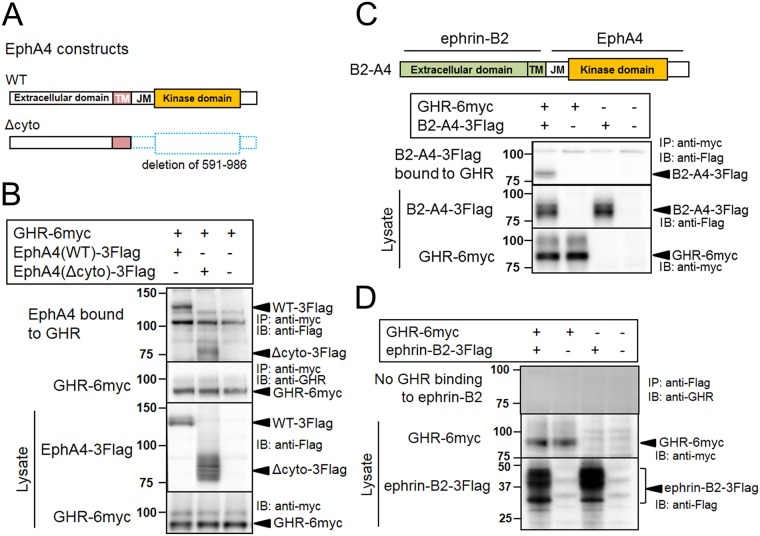
Interaction of EphA4 with GHR. (A) Schemes of wild-type (WT) and a cytoplasmic deletion mutant of EphA4. The numbers represent the amino acid numbers counting from the one encoded by the initiation codon. TM, transmembrane domain; JM, juxtamembrane domain. Dotted blue rectangles indicate the deleted amino-acid regions. (B) Binding between the extracellular domains of EphA4 and GHR. EphA4(WT)-3Flag or EphA4(Δcyto)-3Flag was transiently co-expressed with GHR(WT)-6myc in HEK293T cells. These proteins were subjected to co-immunoprecipitation (IP), fractionation with SDS-PAGE, and detection by immunoblotting (IB) using the indicated antibodies. Arrowheads indicate the molecules shown in the figure. The other bands are regarded as non-specific bands, compared with the result without EphA4 expression (see also S1 Fig). (C) Binding between the cytoplasmic domains of EphA4 and GHR. GHR(WT)-6myc and the chimeric protein in which the cytoplasmic domain of EphA4 was fused to the extracellular domain of ephrin-B2 (B2-A4-3Flag) were co-expressed in HEK293T cells and their interaction was examined by IB following IP and SDS-PAGE fractionation. The bands at approximately 100 kDa for GHR-6myc transfectants probably correspond to the GHR molecules modified with carbohydrates. (D) Control experiment for (C) showing no interaction between GHR and ephrin-B2. Ephrin-B2-3Flag was transiently co-expressed with GHR(WT)-6myc in HEK293T cells and their interaction was examined as described in (C). Some of the molecules that have extracellular domains (EphA4, ephrin-B2, and B2-A4) show broad bands, with the upper bands represented by the carbohydrate-modified proteins. All the IP and IB studies in this figure were repeated 3 times to confirm reproducibility, and representative results are shown.

We previously reported that EphA4 forms a complex with the known assembly of GHR/JAK2/STAT5B, leading to the GH- and ephrin-mediated activation of STAT5B through JAK2-dependent and JAK2-independent pathways, which in combination enhance the expression of *IGF1* mRNA [19]. However, it is not clear which region of the EphA4 molecule is responsible for binding to each of these proteins. Our binding evidence for EphA4 with these individual proteins using MEF, in which specific proteins were separately knocked out or knocked down [19], renders reasonable the immunoprecipitation and immunoblotting studies of the exogenously expressed proteins. First of all, to clarify the molecular regions of EphA4 responsible for its interaction with GHR, we examined GHR(WT) binding using EphA4(WT) and EphA4(Δcyto), a cytoplasmic domain deletion mutant (Fig 1A). Co-expression studies in HEK293T cells revealed that both EphA4(WT) and EphA4(Δcyto) can bind to GHR(WT), suggesting that the EphA4 extracellular domain binds to GHR (Fig 1B). To study whether the intracellular domain of EphA4 can bind to GHR, we used a chimeric ephrin-B2/EphA4 molecule (B2-A4) that has the extracellular and transmembrane domains of ephrin-B2 and the cytoplasmic domain of EphA4 (Fig 1C). Co-expression of GHR(WT) with this chimeric ephrin-B2/EphA4 revealed their binding (Fig 1C). Ephrin-B2 itself, when used as control, did not bind to GHR (Fig 1D). Taken together, EphA4 and GHR bind through both their extracellular and cytoplasmic domains.

### JAK2 binds to the kinase domain of EphA4

**Fig 2:**
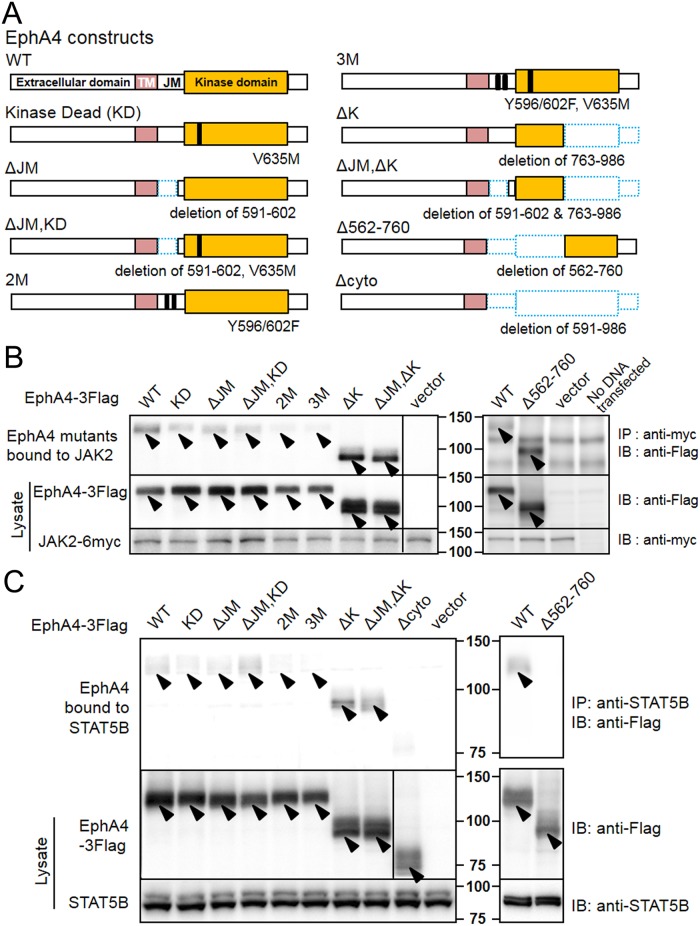
Interaction of EphA4 with JAK2 and STAT5B. (A) Schemes of WT and various cytoplasmic mutants of EphA4. The numbers represent the amino acid numbers counting from the one encoded by the initiation codon. TM, transmembrane domain; JM, juxtamembrane domain. Dotted blue rectangles indicate the deleted amino-acid regions. (B) Binding between EphA4 mutants and JAK2. EphA4(WT) and mutants, which were tagged with 3Flag, were transiently co-expressed with JAK2(WT)-6myc in HEK293T cells. Cell lysates were used for IP and/or IB studies with the indicated antibodies. Transfection with DNA for vector alone (vector) in place of DNA for EphA4 mutants was used as a control. Cells that were treated for transfection with no DNA (No DNA transfected) were used to exclude contamination of any transfected DNA with other DNA, showing that plasmid DNA for vector or JAK2-6myc does not change the IP and IB results shown in the figure. Arrowheads indicate the molecules expressed after transfection. The other bands are regarded as non-specific bands in comparison with the result of vector transfection (see also S1 Fig). The same experiments were repeated 3 times, showing similar results. (C) Binding between EphA4 mutants and STAT5B. EphA4(WT) and mutants, which were tagged with 3Flag, were transiently co-expressed with STAT5B(WT)-6myc in HEK293T cells. Cell lysates were used for IP and/or IB studies with the indicated antibodies. The lines drawn between the lanes are to indicate that a lane was omitted due to a technical error in loading samples on the gel. All samples were fractionated by SDS-PAGE at the same time and immunoblotted on the same membrane. The same experiments were repeated twice, showing essentially the same results.

Next, to determine which region of the EphA4 molecule binds to JAK2, we co-expressed WT and mutants of EphA4 with JAK2(WT) in HEK293T cells. The EphA4 mutants are shown in a scheme in Fig 2A. Kinase dead (KD) EphA4 has a V635M mutation in the ATP binding region of the kinase domain; ΔJM has a deletion of amino acids 591–602 in the juxtamembrane (JM) domain; ΔJM,KD has a deletion of amino acids 591–602 and V635M; 2M has two mutations, Y596F and Y602F; 3M has the mutations of 2M plus V635M; ΔK has a deletion of the carboxy-terminal region of the cytoplasmic domain; ΔJM,ΔK has a combination of the deletions of ΔJM and ΔK; Δ562–760 has a deletion of the amino-terminal region (562–760) of the cytoplasmic domain; and Δcyto has a total deletion of the cytoplasmic domain (amino acids 591–986). Phosphorylation of the tyrosine residues at 596 and 602, which are located within the JM domain, is required for the activation of EphA4; therefore, their mutation from tyrosine to phenylalanine strongly reduces EphA4 activation [26]. The V635M mutation also inhibits EphA4 activation by disrupting its tyrosine kinase activity [13]. Immunoblotting following immunoprecipitation revealed that JAK2 bound to EphA4(WT), EphA4(ΔJM), EphA4(KD), and EphA4(ΔJM,KD) and barely bound to EphA4(2M) and EphA4(3M) (Fig 2B left panel). These findings might be explained by the ability of each molecule to take on an active conformation. We and others have shown that EphA4(ΔJM) behaves as an EphA4(WT) molecule with the tyrosine-phosphorylated JM domain, and presents a conformation exposing the kinase domain to substrates [13,26,27]. The present study also showed that the carboxy-terminal deletion mutants of EphA4, EphA4(ΔK) and EphA4(ΔJM,ΔK), strongly bound to JAK2 (Fig 2B left panel), suggesting that the amino-terminal portion of the EphA4 kinase domain is responsible for this interaction. The carboxy-terminal portion of the EphA4 kinase domain is known to block the access of substrates to the amino-terminal region of the kinase domain in an inactive closed conformation, and this conformation can be released by stimulation with a ligand [27]. The exposed amino-terminal region of the kinase domain is known to interact with other molecules [27]. Thus, we speculated that deletion of the large carboxy-terminal portion might induce EphA4 to take an open conformation for these molecules.

We then examined whether the EphA4 carboxy-terminal region of the cytoplasmic domain is also responsible for binding to JAK2. We constructed a deletion mutant of the JM domain and the amino-terminal region of the EphA4 kinase domain, EphA4(Δ562–760) (Fig 2A). When this mutant was co-expressed with JAK2, they bound to each other (Fig 2B right panel). All of these findings suggest that JAK2 binds to the EphA4 cytoplasmic domain at multiple sites.

### STAT5B binds to the kinase domain of EphA4

In our previous report, we showed that STAT5B bound to EphA4 and was released after the activation of EphA4 with ephrin-A1 in a JAK2 expression-independent manner [19]. To determine which region of the EphA4 molecule binds to STAT5B, we co-expressed WT and mutants of EphA4 with STAT5B(WT) in HEK293T cells (Fig 2C). Immunoblotting following immunoprecipitation showed results similar to the binding between EphA4 and JAK2. STAT5B bound to EphA4(WT), EphA4(KD), EphA4(ΔJM), and EphA4(ΔJM,KD), which can take on a more or less active conformation by overexpression (Fig 2C left panel). However, STAT5B barely bound to EphA4(2M) and EphA4(3M), both of which stay in an inactive conformation due to mutation of the JM domain [13,27]. STAT5B strongly bound to EphA4(ΔK) and EphA4(ΔJM,ΔK), the carboxy-terminal kinase-deletion mutants that are supposed to have an exposed amino-terminal region of the kinase domain [27]. Further study revealed that EphA4(Δ562–760), which has a deletion of the EphA4 JM domain and the amino-terminal kinase domain, did not retain the STAT5B binding capability of the WT protein (Fig 2C right panel). EphA4(Δcyto) did not bind to STAT5B, as expected. These findings suggest that STAT5B binds to the amino-terminal region of the EphA4 kinase domain.

### EphA4 binds to the carboxy-terminal region of JAK2

**Fig 3:**
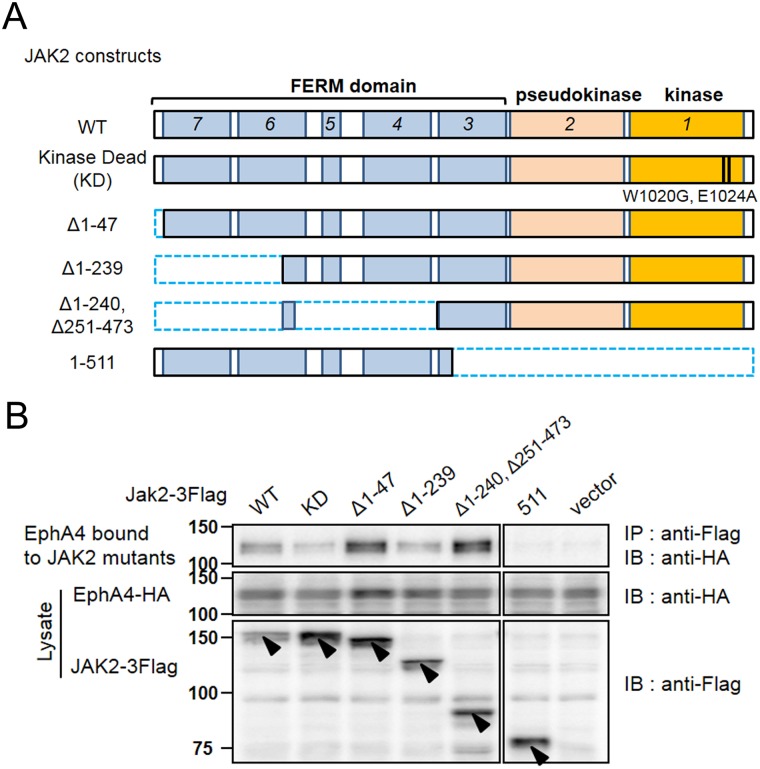
Interaction between JAK2 mutants and EphA4. (A) Schemes to show JAK2(WT) and its deletion mutants. Dotted blue rectangles indicate the deleted amino-acid regions. (B) JAK2(WT) and mutants, which were tagged with 3Flag, were transiently co-expressed with EphA4(WT)-HA in HEK293T cells, and the cells were exposed to 0.5 μg/mL oligomerized ephrin-A1 for 75 min. Cell lysates were used for IP and/or IB studies with the indicated antibodies. All samples were fractionated by SDS-PAGE at the same time and immunoblotted on the same membrane, except that 1 lane between JAK2(Δ1–240, Δ251–473) and JAK2(511) was omitted due to a technical error. Arrowheads indicate the JAK2 mutants expressed in the cells. The other bands are regarded as non-specific bands in comparison with the result of control vector transfection (see also S1 Fig). The same experiments were repeated twice, showing similar results. Deletion of the carboxy-terminal half of the JAK2 molecule abolished the binding of JAK2 to EphA4.

It is established that the amino-terminal region of JAK2 binds to GHR [23,28–30]. We then examined which region of JAK2 is responsible for binding to EphA4(WT) using JAK2(WT) and its mutants (Fig 3A). Co-expression and immunoprecipitation experiments revealed the following findings. Unless the cells were stimulated with ephrin, the binding of EphA4(WT) to JAK2(WT) or mutants was so weak that we could barely detect the binding by immunoblotting (data not shown). Under stimulation with ephrin-A1, JAK2(WT) bound to EphA4 more strongly than JAK2(KD), and the JAK2 amino-terminal deletion mutants (Δ1–47, Δ1–239, Δ1–240, and Δ251–473) mostly bound to EphA4 (Fig 3B). However, a large deletion of the carboxy-terminus, JAK2(1–511), abolished this binding. These findings suggest that the carboxy-terminal region of the whole JAK2 molecule is mainly responsible for binding to EphA4.

Thus, the JAK2 molecule appears to bind to GHR and EphA4 using both ends of the molecule, that is, the amino-terminus to GHR and the carboxy-terminus to EphA4. This differential binding may play an important role in downstream signal transduction through STAT5B via 2 different receptors. We then proceeded to study the activation/phosphorylation pathways mediated by both of these receptors.

### EphA4 phosphorylates GHR in a JAK2-independent manner

**Fig 4:**
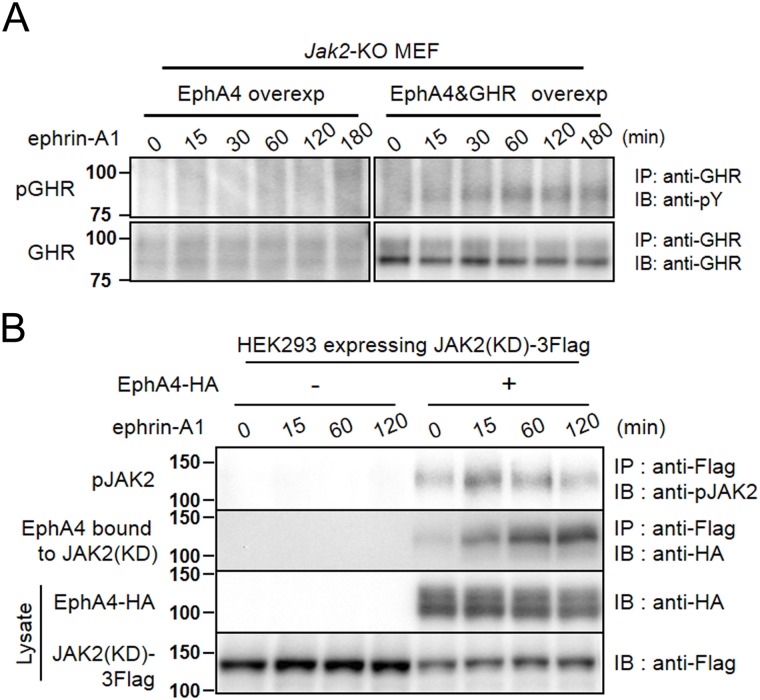
Phosphorylation of GHR and JAK2 by ligand-activated EphA4. (A) GHR phosphorylation by EphA4 in the absence of JAK2. *Jak2*-KO mouse embryonic fibroblasts (MEF) retrovirally overexpressing EphA4 (EphA4 overexp) and those overexpressing EphA4 and Ghr (EphA4&GHR overexp) were pre-incubated with serum-free medium overnight and incubated with ephrin-A1 (0.5 μg/mL) for the time shown. Cell lysates were used for IP and/or IB studies with the indicated antibodies. Anti-pY: anti-phosphotyrosine antibody. The same experiments were repeated 3 times, showing the same results. (B) JAK2 phosphorylation by EphA4. HEK293T cells expressing JAK2(KD)-Flag with or without EphA4(WT)-HA were preincubated with serum-free medium for 6 h, then stimulated with ephrin-A1 (0.5 μg/mL) for the time shown. Cell lysates were detected by IB and/or IP with the indicated antibodies. The same experiments were repeated 3 times, showing the same results.

Our previous studies demonstrated that the binding between GHR and EphA4 appeared to be strengthened by the activation of EphA4 with ephrin-A1 [19]; therefore, we examined whether activated EphA4 phosphorylates GHR in a JAK2-independent manner (Fig 4A). We overexpressed EphA4 alone or together with GHR in *Jak2*-knockout (KO) MEF using a retrovirus vector. Following stimulation with ephrin-A1, endogenous GHR was barely phosphorylated, but this activation became clear by over-expression of GHR. EphA4 phosphorylated GHR in the absence of JAK2. These findings suggest that GHR can be a direct target of EphA4 and partly explain the crosstalk between EphA4 and GHR.

### EphA4 phosphorylates JAK2

We previously reported that activated JAK2 phosphorylates EphA4 [19]. We studied the signal transduction pathway from EphA4 to JAK2 in more detail. To examine whether EphA4 can phosphorylate JAK2 directly, JAK2(KD), a kinase-dead mutant (Fig 3A) [31], and EphA4(WT) were co-expressed in HEK293T cells (Fig 4B). Exposure to ephrin-A1 induced JAK2(KD) phosphorylation in these HEK293T cells, with a peak at 15 min. We also detected stronger binding between JAK2(KD) and EphA4 after stimulation with ephrin-A1 than before stimulation. These findings suggest that EphA4 activates not only GHR, as shown above, but also JAK2 by direct phosphorylation.

### GHR expression is essential for direct STAT5B phosphorylation by EphA4

**Fig 5:**
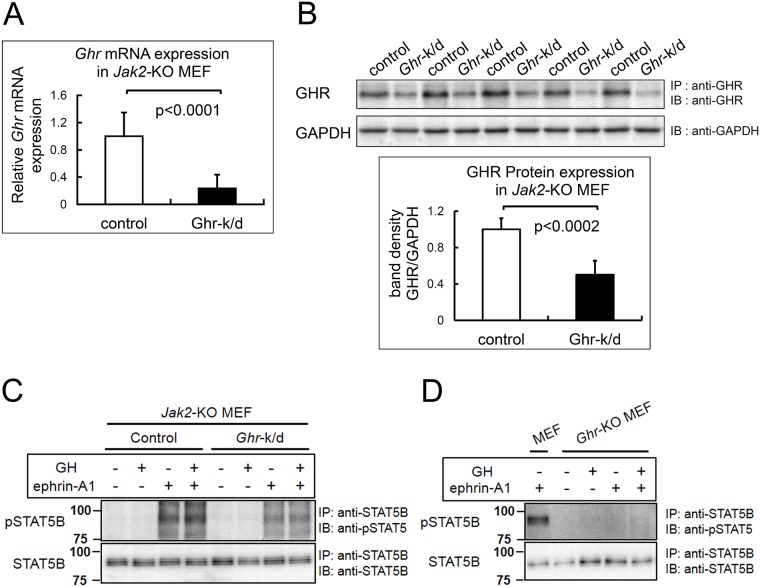
Requirement of GHR for EphA4-mediated STAT5B phosphorylation. (A) *Ghr* mRNA expression in *Ghr*-knockdown (*Ghr*-k/d) *Jak2*-KO MEF. *Jak2*-KO MEF were transduced with lentivirus carrying either *Ghr* shRNA (*Ghr*-k/d) or control shRNA at an MOI of 10. The *Ghr* mRNA expression levels were measured in 8 independent MEF clones using quantitative real-time RT-PCR. Data are shown as mean ± standard deviation (SD) values of the 8 samples. (B) GHR protein expression levels. Cells from one of the MEF clones used for the following experiments were lysed and used for western blotting to quantify GHR protein expression. The results of 5 independent studies are shown in the upper panel, and their band densities are presented as mean ± SD values in the lower panel. (C) Effect of GHR knockdown on JAK2-independent STAT5B phosphorylation. *Jak2*-KO MEF with or without *Ghr*-k/d were pre-incubated in serum-free medium overnight (no ligands) and exposed to GH and/or ephrin-A1. The cells were exposed to ephrin-A1 (500 ng/mL) for 75 min, and to GH (500 ng/mL) for 30 min. In case of exposure to both GH and ephrin-A1, the cells were pre-exposed to ephrin-A1 for 45 min and then exposed to GH for 30 min. Cell lysates were used for IP and IB studies with the antibodies indicated in the figure. The same experiments were repeated 3 times, showing similar results. The amount of phosphorylated STAT5B (pSTAT5B) was reduced in the *Ghr*-k/d cells. (D) Effect of GHR KO on STAT5B phosphorylation. The same experiments as in (C) were carried out in *Ghr*-KO MEF. The same experiments were repeated 3 times, showing the same results. The complete absence of GHR (*Ghr*-KO) abolished STAT5B phosphorylation.

To examine how GHR expression affects JAK2-independent EphA4/STAT5B signaling, we knocked down *Ghr* by infecting *Jak2*-KO MEF with a lentivirus that expresses *Ghr* shRNA (Santa Cruz; catalog #sc-40016-v) or control shRNA (Santa Cruz; catalog #sc-108080). *Jak2*-KO MEF with knocked-down *Ghr* (*Ghr*-k/d & *Jak2*-KO MEF) showed reduced expression of *Ghr* mRNA and GHR protein by approximately 80% and 50% (Fig 5A and 5B), respectively, compared with control *Jak2*-KO MEF. In these cells, we studied STAT5B phosphorylation following stimulation with ephrin-A1 and/or GH. *Ghr*-k/d & *Jak2*-KO MEF showed lower levels of STAT5B phosphorylation in response to ephrin-A1 stimulation compared with control *Jak2*-KO MEF (Fig 5C), suggesting that ephrin-A1-induced JAK2-independent STAT5B phosphorylation depends on the expression of GHR. Then, a similar approach was used to examine STAT5B phosphorylation in *Ghr*-KO MEF (Fig 5D). Here, in the complete absence of GHR, no STAT5B phosphorylation was detectable in response to ephrin-A1 stimulation, further supporting the theory that ephrin-mediated STAT5B phosphorylation requires GHR expression.

### Ligand-activated EphA4 enhances the nuclear translocation of STAT5B

**Fig 6:**
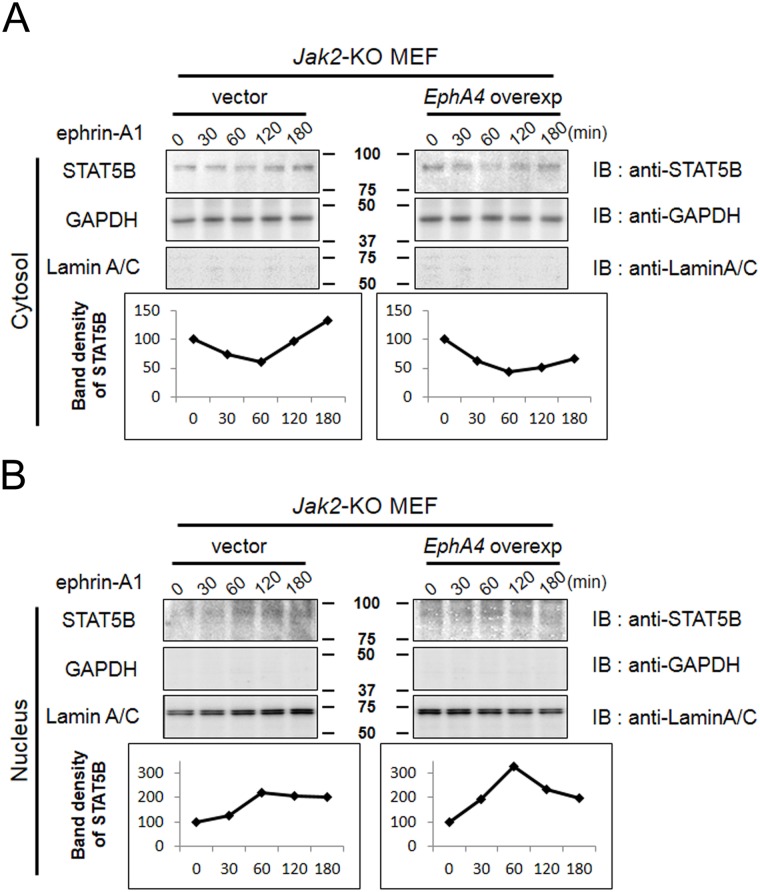
Ephrin A1-mediated translocation of STAT5B from the cytosol to the nucleus. *Jak2*-KO MEF were pre-incubated in serum-free medium overnight and exposed to ephrin-A1 (500 ng/mL). After incubation with ephrin-A1 for the time indicated in the figure, Jak2-KO MEF were harvested from the dish using a scraper, and cytosolic (A) and nuclear (B) extracts were separated as described in the Methods section. GAPDH was used as an indicator for cytosolic extracts and LaminA/C for nuclear extracts. Cytosolic and nuclear extracts were immunoblotted with anti-STAT5B, anti-GAPDH, and anti-LaminA/C antibodies, and band density was quantified. The same experiments were repeated twice, showing similar results.

We have shown here that EphA4 can phosphorylate STAT5B, but it is not yet clear whether the nuclear translocation of phosphorylated STAT5B is enhanced by EphA4. We examined the nuclear localization of STAT5B by nuclear-cytosol fractionation in *Jak2*-KO fibroblasts and EphA4-overexpressing *Jak2*-KO fibroblasts. The cells were exposed to ephrin-A1 for periods of up to 180 min and harvested for nuclear-cytosol fractionation. Nuclear fractionation was confirmed by LaminA/C expression, while cytosol fractionation was confirmed by GAPDH expression. This analysis revealed that the amount of STAT5B in the cytosol decreased to a minimum at 60 min of ephrin-A1 stimulation (Fig 6A) and its level in the nucleus increased to a maximum at 60 min in both cell lines (Fig 6B). These results suggest that STAT5B translocates from the cytosol to the nucleus with a peak at 60 min of ephrin-A1 stimulation.

### JAK2 activated by GH does not phosphorylate STAT5B molecules that are bound to EphA4

**Fig 7:**
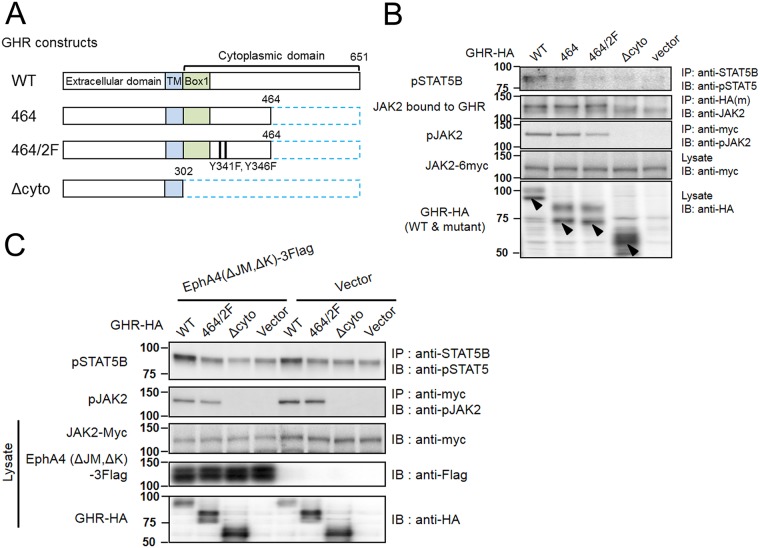
JAK2 activated by GH does not phosphorylate EphA4-bound or free STAT5B. (A) Structure of GHR WT and mutants. The numbers represent the amino acid numbers counting from the initiation codon. “Box1,” which is the proline-rich region of the cytoplasmic domain, is necessary for GH-dependent association with JAK2 and the following activation of JAK2 [32]. TM: transmembrane domain. The numbers represent the amino acid numbers counting from the one encoded by the initiation codon. (B) Binding of JAK2 with GHR (WT and mutants) and phosphorylation of STAT5B. GHR mutants tagged with HA were co-expressed with JAK2-myc in HEK293T cells and the cells were stimulated with GH for 15 min. GHR(464/2F) bound to and activated JAK2, but did not phosphorylate STAT5B. (C) Phosphorylation of STAT5B bound to kinase-deleted EphA4 by GH-activated JAK2. GHR(WT), (464/2F), or (Δcyto) was co-expressed in HEK293T cells with EphA4(ΔJM,ΔK)-3Flag, which binds to STAT5B (Fig 2B), then the cells were stimulated with GH for 15 min. The expression of EphA4(ΔJM,ΔK)-3Flag did not alter the extent of STAT5B phosphorylation as compared with the control (vector). The cell lysates were used for IP and/or IB with the indicated antibodies. Arrowheads indicate the molecules expressed following transfection. The other bands are regarded as non-specific bands in comparison with the result of vector transfection (see also S1 Fig). GHR molecules are detected as double or diffuse bands with the upper bands representing carbohydrate-modified molecules. All the experiments in (B) and (C) were repeated twice, showing similar results.

To examine whether GH-activated JAK2 can phosphorylate STAT5B bound to EphA4, we constructed 3 GHR mutants, which were homologous to the mutants reported by Smit et al. [32]. GHR(464) has a deletion of the carboxy-terminal 197 amino acids; GHR(464/2F) has a deletion of the carboxyl-terminal 197 residues and substitution of 2 tyrosine residues with phenylalanine at positions 341 and 346; and GHR(Δcyto) lacks the entire carboxyl-terminal 349 residues of the cytoplasmic domain (Fig 7A). When GHR(WT) or these mutants were co-expressed with JAK2 in HEK293T cells, which barely express JAK2, and incubated with GH for 15 min, both GHR(464) and GHR(464/2F) equally bound to JAK2. STAT5B phosphorylation was different in that GHR(464) activated STAT5B slightly over the basal level, but GHR(464/2F) did not (Fig 7B). These findings suggest that GHR(464/2F) has the capacity to bind to JAK2 without retaining the capability of STAT5B phosphorylation, probably due to a lack of temporary binding of STAT5B to GHR for phosphorylation by JAK2. These findings replicate those of a previous report [32].

Then, we examined if GH signaling can result in the phosphorylation of EphA4-bound STAT5B via JAK2 activation. EphA4(ΔJM,ΔK) has no kinase activity but can still bind strongly to STAT5B (Fig 2B) and can bind to GHR (Fig 1) at least through the extracellular domain. Cells co-expressing GHR(WT) and EphA4(ΔJM,ΔK) in the presence of exogenous JAK2 expression showed no change in STAT5B phosphorylation compared with those expressing GHR(WT) alone after stimulation with GH for 15 min (Fig 7C). GHR(464/2F) retained the capability of binding to and activating JAK2, but not the capability of inducing STAT5B phosphorylation. It did not show further STAT5B phosphorylation over the basal level even in the presence of the exogenous expression of JAK2 and EphA4(ΔJM,ΔK). All of these findings imply that neither free STAT5B nor STAT5B bound to EphA4 in the cytosol is the phosphorylation target of JAK2 bound to GHR.

### Various ephrins can stimulate STAT5B signaling

**Fig 8:**
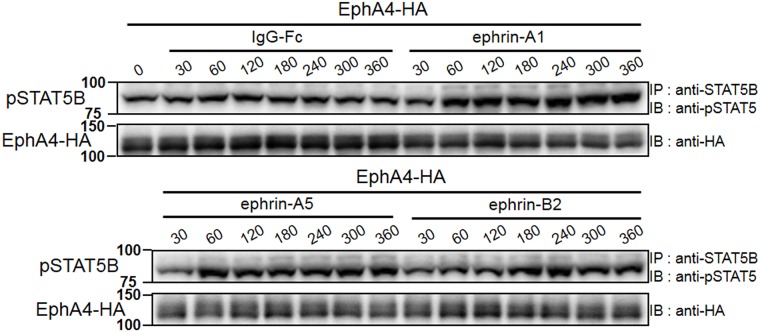
Effect of various ephrins on EphA4/GHR/JAK2/STAT5B signaling. (A) EphA4-HA was expressed in HEK293T cells, then stimulated with oligomerized ephrin-A1-Fc, ephrin-A5-Fc, ephrin-B2-Fc, or IgG-Fc alone as shown in the Materials and Methods for the time indicated. Cell lysates were fractionated with SDS-PAGE. pSTAT5B was detected by IB following IP using the antibodies shown, and EphA4-HA was detected with an anti-HA antibody. The same experiments were repeated 3 times, showing similar results.

We have so far used only ephrin-A1 as a ligand for stimulating EphA4, which can bind to several ephrins of 2 different classes, A and B [18]. To examine whether other ephrins affect EphA4/GHR/JAK2/STAT5B signaling, we overexpressed EphA4 in HEK293T cells and stimulated it with ephrin-A1, ephrin-A5, or ephrin-B2 for up to 360 min (Fig 8). These ligands are composed of their extracellular domains fused to a human IgG-Fc fragment. Since ephrins effectively stimulate Eph receptors after oligomerization, IgG-Fc-fused ephrins are oligomerized using an anti-IgG-Fc antibody before application, and oligomerized IgG-Fc is usually used as a control for ephrins [33]. STAT5B phosphorylation was enhanced with a peak at 60–180 min of exposure to ephrin-A1 or ephrin-A5 and a peak at 180 min of ephrin-B2 stimulation. IgG-Fc itself did not enhance STAT5B phosphorylation. These results clearly show that not only ephrin-A1 but also other ephrins can activate STAT5B, suggesting that EphA4 can be used ubiquitously for the activation of STAT5B.

**Fig 9:**
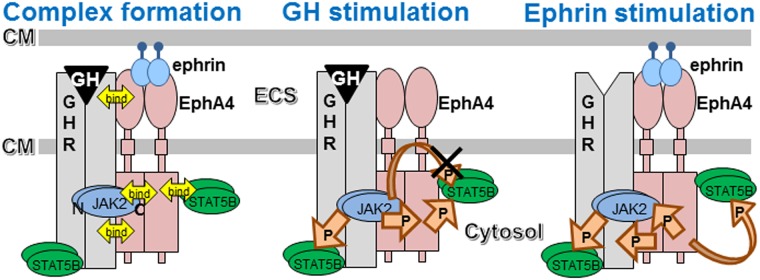
Molecular interaction schemes between GH/GHR and ephrin/EphA4 signaling. Interactive binding of the molecules participating in the GH/GHR and ephrin/Eph signal transduction pathways is shown in the left-side panel. When both GH and ephrin were applied to the cells, GHR and EphA4 bind to each other through both extracellular and intracellular domains. Then, JAK2 and STAT5B are attracted to the complex: the amino-terminal region of JAK2 to GHR, the carboxy-terminal region to EphA4, and STAT5B to the EphA4 kinase domain in addition to the known binding of STAT5B to the cytoplasmic domain of GHR. The middle panel shows the actions of JAK2 kinase and EphA4 kinase in response to GH. The activated complex-bound JAK2 phosphorylates both EphA4 and STAT5B that are bound to GHR, but not STAT5B bound to EphA4. EphA4 kinase activity is also enhanced by JAK2-mediated phosphorylation leading to the enhanced phosphorylation of STAT5B that is bound to EphA4. The right panel indicates the actions of EphA4 kinase and JAK2 kinase in response to ephrin. The activated EphA4 kinase phosphorylates JAK2, GHR, and STAT5B bound to EphA4. The activated JAK2 is supposed to activate STAT5B bound to GHR subsequently. This ephrin-initiated signaling requires the presence of GHR. The molecular explanation for this requirement is unknown. P, phosphorylation; N, amino-terminal domain; C, carboxy-terminal domain; CM, cell membrane; ECS, extracellular space.

In conclusion, we report the molecular interactions of the GHR/EphA4/JAK2/STAT5B signaling pathway. GHR and EphA4 bind to each other through both the extracellular and cytoplasmic domains, as shown in the scheme in Fig 9. The amino-terminal region of the EphA4 kinase domain binds to both JAK2 and STAT5B. The region of JAK2 responsible for this binding is the carboxy-terminal portion, in contrast to the amino-terminal portion of JAK2 that is known to bind to GHR [28]. When stimulated with ligands, JAK2 kinase downstream of GHR crosstalks with EphA4 via mutual phosphorylation, leading to the enhanced activation of STAT5B. STAT5B bound to EphA4 appears not to be the substrate for JAK2 downstream of GH/GHR signaling. Interestingly, the activated ephrin/EphA4 signaling pathway requires the presence of GHR expression for STAT5B activation, suggesting that GHR is the key molecule regulating this signal transduction pathway.

## Supporting information

S1 Fig Control experiments for immunoprecipitation and immunoblotting studies. (A) HEK293T cells were transfected with a pCDNA3.1 eukaryotic expression vector itself or the vector expressing EphA4-3Flag or GHR-HA. Cell lysate (200 μg) from each transfectant was immunoprecipitated (IP) with the antibodies shown and immunoblotted (IB) with the antibodies indicated. The right panel shows the IP & IB study in which only protein A-agarose without any antibody was used for IP, and only the secondary antibody (anti-mouse IgG-HRP) without primary antibody was used for IB. (B) The same experiments as in (A) except the proteins expressed in the cells, GHR-6myc and GHR-HA, and the antibodies used for IP and IB. (C) The same experiments as in (A) except for the antibodies used for IP and IB. (D) The same experiments as in (A) except for the antibodies used for IP and IB. (E) The same experiments as in (A) except for the protein expressed in the cells, JAK2-6myc, and the antibodies used for IP and IB. The right panel shows the re-blot picture of the left panel. IP with and without each antibody is indicated as + and–, respectively. The antibody used for IB is shown at the bottom of the data. Arrowheads indicate the protein molecules detected. Ab indicates the antibody used for IP and detected by the subsequent IB.
(TIF)
