# Supplementary figures and images for: Molecular interactions of EphA4, growth hormone receptor, Janus kinase 2, and signal transducer and activator of transcription 5B
Source: PLoS One. 2017 Jul 7;12(7):e0180785. doi: 10.1371/journal.pone.0180785 (PMC5501605; doi:10.1371/journal.pone.0180785)

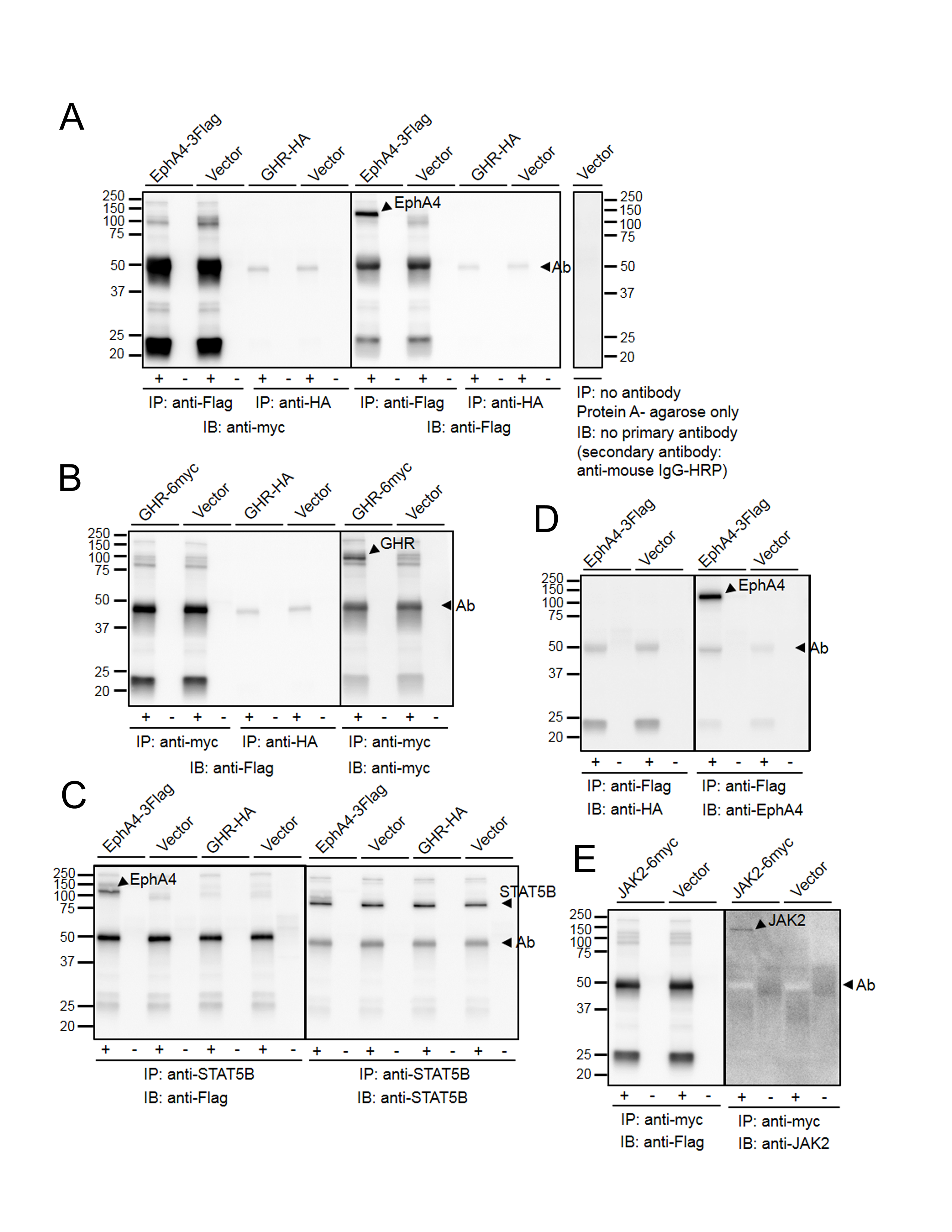

Supplement: S1 Fig — (A) HEK293T cells were transfected with a pCDNA3.1 eukaryotic expression vector itself or the vector expressing EphA4-3Flag or GHR-HA. Cell lysate (200 μg) from each transfectant was immunoprecipitated (IP) with the antibodies shown and immunoblotted (IB) with the antibodies indicated. The right panel shows the IP & IB study in which only protein A-agarose without any antibody was used for IP, and only the secondary antibody (anti-mouse IgG-HRP) without primary antibody was used for IB. (B) The same experiments as in (A) except the proteins expressed in the cells, GHR-6myc and GHR-HA, and the antibodies used for IP and IB. (C) The same experiments as in (A) except for the antibodies used for IP and IB. (D) The same experiments as in (A) except for the antibodies used for IP and IB. (E) The same experiments as in (A) except for the protein expressed in the cells, JAK2-6myc, and the antibodies used for IP and IB. The right panel shows the re-blot picture of the left panel. IP with and without each antibody is indicated as + and–, respectively. The antibody used for IB is shown at the bottom of the data. Arrowheads indicate the protein molecules detected. Ab indicates the antibody used for IP and detected by the subsequent IB. (TIF) [file pone.0180785.s001.tif]
